# Crystal structure of Cs_2_GdNb_6_Cl_15_O_3_ in the structural evolution of niobium oxychlorides with octa­hedral Nb_6_-cluster units

**DOI:** 10.1107/S205698902300871X

**Published:** 2023-10-10

**Authors:** Saronom Silaban, Maefa Eka Haryani, Fakhili Gulo, Christiane Perrin

**Affiliations:** a Department of Chemistry, Medan State University, Medan 20221, Indonesia; b Department of Chemistry Education, Sriwijaya University, Inderalaya, Ogan Ilir 30662, South Sumatra, Indonesia; c Study Program of Environmental Science, Postgraduate Program, Sriwijaya University, Palembang 30139, South Sumatra, Indonesia; d Institut de Chimie de Rennes, Laboratoire de Chimie du Solide et Inorganique Moleculaire, UMR 6511, CNRS-Université de Rennes 1, Avenue du Général Leclerc, 35042 Rennes Cedex, France; Vienna University of Technology, Austria

**Keywords:** cluster compound, crystal structure, valence electron count

## Abstract

Cs_2_GdNb_6_Cl_15_O_3_, dicaesium gadolinium hexa­niobium penta­deca­chloride trioxide, was synthesized by solid-state reactions starting from a stoichiometric mixture of CsCl, Gd_2_O_3_, Nb, NbCl_5_, and Nb_2_O_5_. The crystal structure is based on octa­hedral Nb_6_ cluster units (point group symmetry 3.2) with composition [(Nb_6_Cl^*i*^_9_O^*i*^_3_)Cl^*a*^_6_]^5–^ where *i* and *a* denote inner and outer ligands. Cs_2_GdNb_6_Cl_15_O_3_ exhibits 14 valence electrons per cluster unit. The cluster units are linked to each other by Cs^I^ and Gd^III^ atoms, whereby Cs^I^ (site symmetry 3..) is 12-coordinated by six Cl^*i*^ and six Cl^*a*^ ligands belonging to six neighboring cluster units and Gd^III^ (site symmetry 3.2) is 9-coordinated by three O^*i*^ and six Cl^*i*^ ligands belonging to three adjacent cluster units. The arrangement of cluster units corresponds to a stacking of …*AA*′*A*… layers along [001]. Cs_2_GdNb_6_Cl_15_O_3_ is isotypic with Cs_2_UNb_6_Cl_15_O_3_.

## Abstract 2

Cs_2_GdNb_6_Cl_15_O_3_ is an octa­hedral Nb-cluster compound containing three inner ligands of oxygen atoms. Individual Nb_6_ clusters are linked to each other *via* Gd^III^ and Cs^I^ atoms, which exhibit a coordination number of 9 (three O and six Cl ligands) and 12 (twelve Cl ligands).

## 1. Chemical context

Transition-metal clusters have high potentials and synergetic effects in the fields of biotechnology, catalysis, or sensor applications (Nguyen *et al.*, 2022 ▸). The use of these clusters as supra­molecular building units is advantageous because of their unique structural, chemical and physical properties (Zhou & Lachgar, 2007 ▸). For example, charge-transfer (CT) solids with an anti-perovskite crystal structure have been derived from molybdenum cluster units by electro-crystallization (Hiramatsu *et al.*, 2015 ▸), or octa­hedral cluster units of niobium have been widely used as raw materials for the preparation of novel compounds with inter­esting structures and magnetic properties (Naumov *et al.*, 2003 ▸; Zhang *et al.*, 2011 ▸).

A large number of binary, ternary and quaternary niobium compounds with octa­hedral clusters based on the [Nb_6_ *L^i^*_12_ *L^a^*_6_] unit (*L* = halogen or oxygen ligands) have been reported previously (Perrin *et al.*, 2001 ▸). In this cluster unit, the edge of the Nb_6_ octa­hedron is bridged by twelve inner ligands (*L^i^*) while the other six outer ligands (*L^a^*) are located at apical positions (Schäfer & von Schnering, 1964 ▸). The number of electrons involved in the formation of metal–metal bonds in the cluster is called the valence electron count (VEC). The ideal VEC value per cluster is 16 for chloride compounds and 14 for oxide compounds. In chlorides, the cluster units are inter­linked by involving outer ligands (Perrin, 1997 ▸), whereas in oxides, the connectivity between the units is achieved through the inner ligands (Köhler *et al.*, 1991 ▸).

Mixing of halogen and oxygen as ligands for Nb_6_ cluster compounds is a very inter­esting topic for in-depth studies to enrich our knowledge about new materials and their physical and chemical properties. It has been reported that the structural, magnetic and electronic properties of octa­hedral clusters of niobium oxychlorides are influenced by oxygen ligands (Fontaine *et al.*, 2011 ▸). In this respect, preparation and characterization of new oxychloride compounds with octa­hedral Nb_6_ clusters were reported by Perrin *et al.* with one O^*i*^ (Cordier *et al.*, 1996 ▸), two O^*i*^ (Gulo & Perrin, 2000 ▸), three O^*i*^ (Cordier *et al.*, 1994 ▸, 1997 ▸; Gulo & Perrin, 2002 ▸), and six O^*i*^ (Gulo *et al.*, 2001 ▸) ligands per cluster unit. Other oxychloride compounds containing four O^*i*^ (Anokhina *et al.*, 1998 ▸, 2000 ▸) and six O^*i*^ (Anokhina *et al.*, 2001 ▸) ligands per cluster unit are also known.

The niobium oxychloride compound Cs_2_UNb_6_Cl_15_O_3_ was synthesized and structurally characterized many years ago (Cordier *et al.*, 1997 ▸). We have now prepared a related compound with composition Cs_2_GdNb_6_Cl_15_O_3_ and have determined its crystal structure where gadolinium occupies the same position as uranium in the previous compound. In the current communication, the crystal structure, inter­atomic distances, and the role of monovalent and trivalent cations in Cs_2_GdNb_6_Cl_15_O_3_ are compared with other niobium oxychlorides containing octa­hedral Nb_6_ clusters.

## 2. Structural commentary

**Figure 1:**
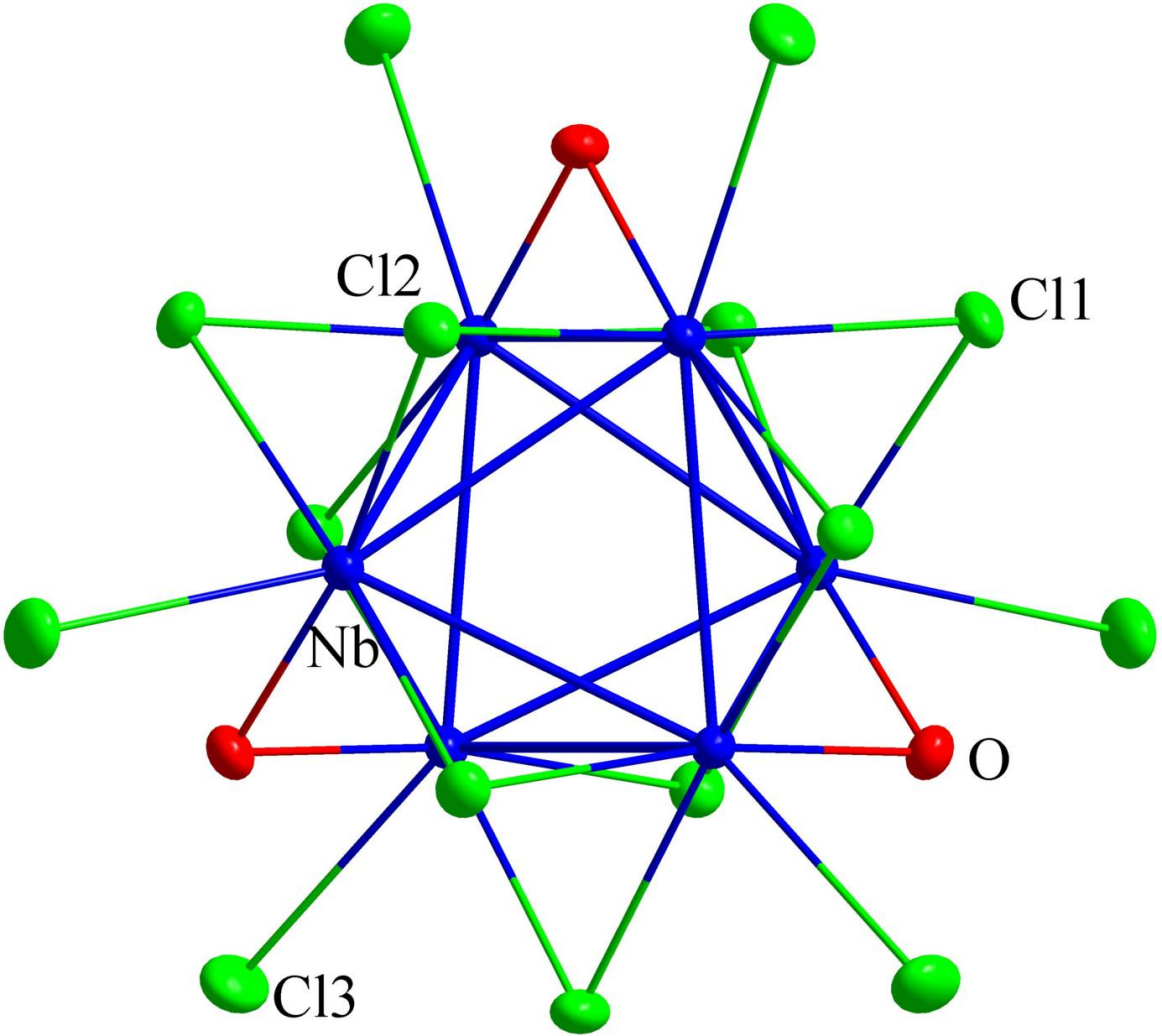
The [(Nb_6_Cl^*i*^_9_O^*i*^_3_)Cl^*a*^_6_]^5–^ unit in the crystal structure of Cs_2_GdNb_6_Cl_15_O_3_. Displacement ellipsoids are shown at the 60% probability level.

The structure of Cs_2_GdNb_6_Cl_15_O_3_ is isotypic with Cs_2_UNb_6_Cl_15_O_3_ and displays the Nb_6_ octa­hedron as the basic cluster motif. The asymmetric unit comprises seven sites: one Cs (site symmetry 3.., multiplicity 4, Wyckoff letter *e*), one Gd (3.2, 2 *c*), one Nb (1, 12 *i*), one O (..2, 6 *h*) and three Cl (Cl1: 6 *h*; Cl2 12 *i*; Cl3: 12 *i*). Six symmetry-equivalent niobium atoms build up the octa­hedral cluster (centered at a position with site symmetry 3.2, 2 *a*). Each niobium atom is surrounded by one oxygen (O) inner-ligand, three chlorine (Cl1 and Cl2) inner-ligands, and one chlorine (Cl3) outer-ligand. Every edge of the Nb_6_ octa­hedron is bridged by a chlorine or oxygen ligand as inner-ligands, and six other chlorine ligands are attached in apical positions as outer ligands, as shown in Fig. 1 ▸. This cluster motif can be written as a developed unit, [(Nb_6_Cl^*i*^_9_O^*i*^_3_)Cl^*a*^_6_]^5–^.

**Figure 2:**
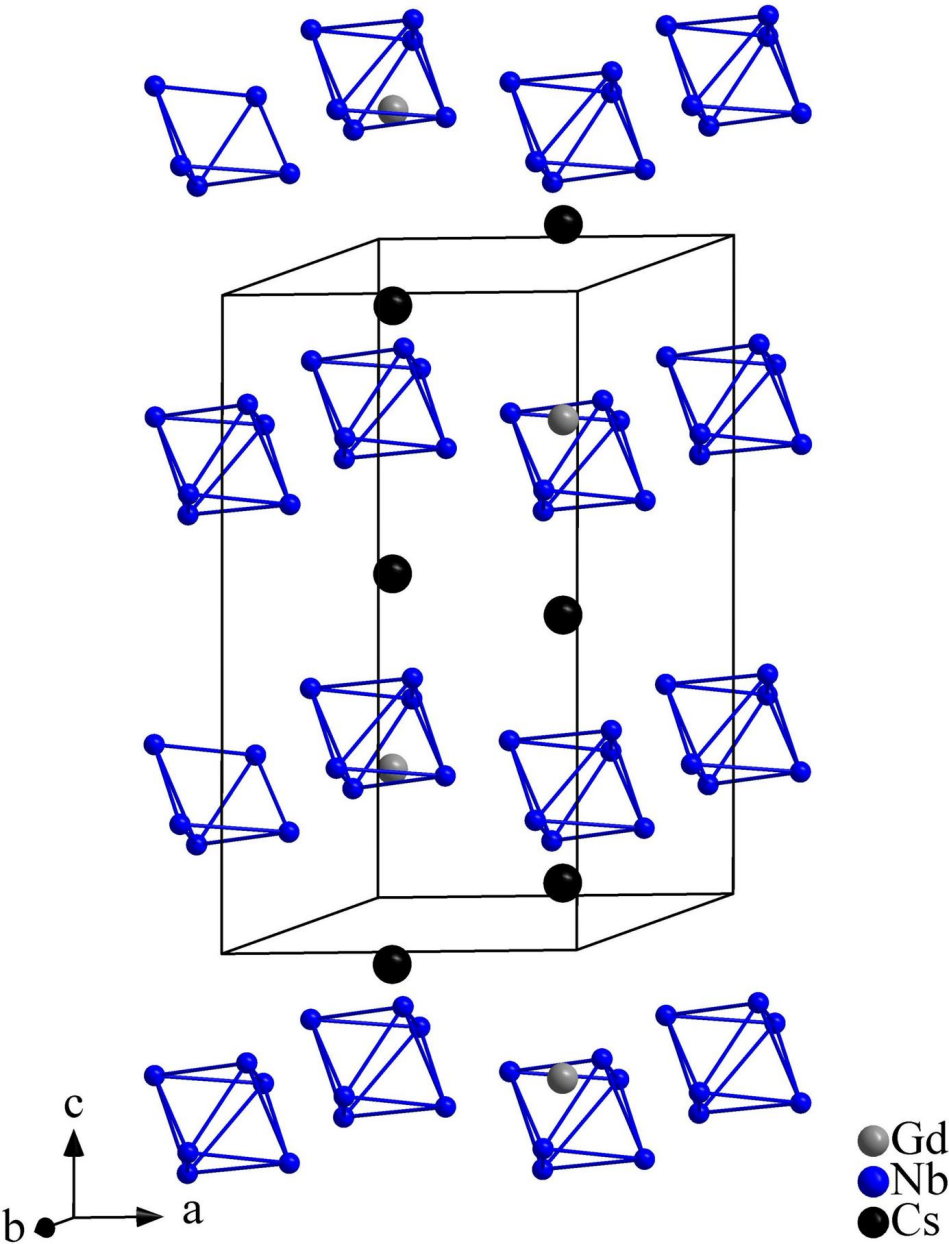
The …*AA*′*A*… stacking of the Nb_6_ clusters in the crystal structure of Cs_2_GdNb_6_Cl_15_O_3_. Cl and O atoms are omitted for clarity

The length of the intra­cluster Nb—Nb bonds range from 2.7686 (5) to 3.0317 (5) Å corresponding to the edge bridged by the O^*i*^ and Cl^*i*^ ligands, respectively; the average bond length is 2.954 Å. Thus, the Nb_6_ octa­hedron undergoes distortions as observed in other niobium oxychloride compounds. The Nb—Nb distances in this compound are significantly shorter than those observed in other compounds containing two or fewer O^*i*^ ligands but are significantly longer than those observed in compounds containing four or more O^*i*^ ligands (Gulo & Perrin, 2012 ▸). In the various oxychloride compounds that have been isolated so far, it seems that an increase in the number of O^*i*^ ligands per formula leads to a decrease in the length of intra­cluster Nb—Nb bonds. This difference is due to a stronger steric effect as observed, for example, in PbLu_3_Nb_6_Cl_15_O_6_ (Gulo *et al.*, 2001 ▸) with six O^*i*^ ligands. Cs_2_GdNb_6_Cl_15_O_3_ has three O^*i*^ ligands per cluster. They are localized at *trans*-inner positions relative to the Nb_6_ cluster, similar to the arrangement of three O^*i*^ ligands in Na_0.21_Nb_6_Cl_10.5_O_3_ where the cluster exhibits point group symmetry 3 (Gulo & Perrin, 2002 ▸). In contrast, the three O^*i*^ ligands in ScNb_6_Cl_13_O_3_ occupy a *cis*-inner position relative to the Nb_6_ octa­hedron to produce a cluster motif with 2 symmetry (Cordier *et al.*, 1994 ▸). In the title compound, the Nb_6_ clusters are arranged in (001) layers with an …*AA*′*A*… stacking along [001] (Fig. 2 ▸).

The Nb—Cl^*i*^ distances vary from 2.4543 (7) Å to 2.4802 (7) Å (average 2.468 Å) while the Nb—Cl^*a*^ bond is longer, 2.5728 (7) Å. In general, the Nb—*L* (*L* = O, Cl) bond lengths in Cs_2_GdNb_6_Cl_15_O_3_ are not significantly different from that of other niobium oxychloride compounds (Naumov *et al.*, 2003 ▸).

In the crystal structure of Cs_2_GdNb_6_Cl_15_O_3_, the [(Nb_6_Cl^*i*^_9_O^*i*^_3_)Cl^*a*^_6_]^5–^ units are inter­connected through the Cs^I^ and Gd^III^ atoms that are located in between the layers of Nb_6_ clusters (Fig. 2 ▸). The existence of such discrete cluster units or the absence of inter­cluster connectivity has also been observed in PbLu_3_Nb_6_Cl_15_O_6_ (Gulo *et al.*, 2001 ▸) and Cs_2_Ti_4_Nb_6_Cl_18_O_6_ (Anokhina *et al.*, 2001 ▸) where Nb_6_-clusters likewise are formed by six symmetry-equivalent Nb atoms in contrast to CsNb_6_Cl_12_O_2_ (Gulo & Perrin, 2000 ▸) where the Nb_6_-octa­hedron is formed by three different Nb atoms. In the crystal structure of the latter, the the cluster units are linked together via bridging O and Cl ligands.

**Figure 3:**
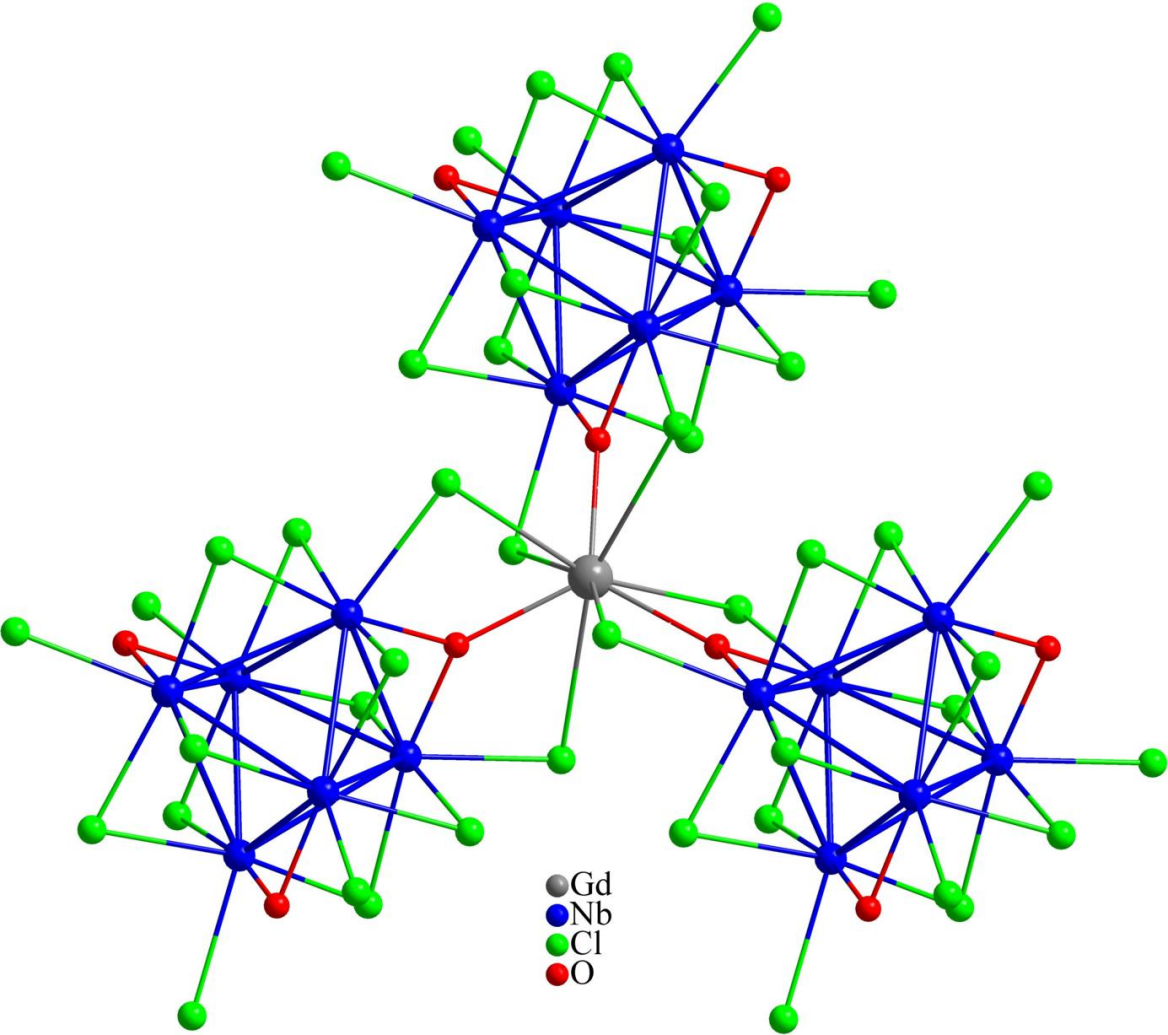
The environment of the Gd^III^ atom in Cs_2_GdNb_6_Cl_15_O_3_.

The Gd^III^ atom in Cs_2_GdNb_6_Cl_15_O_3_ has a coordination number of 9, defined by three O^*i*^ and six Cl^*a*^ ligands provided by three nearby cluster units (Fig. 3 ▸), with bond lengths of Gd—O = 2.322 (3) Å and Gd—Cl = 3.0994 (8) Å. In comparison, in the crystal structure of PbLu_3_Nb_6_Cl_15_O_6_, the Lu^III^ atom is surrounded by only six ligands, *viz*. two O and four Cl atoms, defining Lu_2_Cl_2_ entities (Gulo *et al.*, 2001 ▸). The Nb_6_-clusters in PbLu_3_Nb_6_Cl_15_O_6_ connect to each other *via* these Lu_2_Cl_2_ entities whereby each cluster is surrounded by six Lu_2_Cl_2_ entities, and each of them bridging four adjacent clusters via O and Cl ligands. A related motif is found in Ti_2_Nb_6_Cl_14_O_4_ where Ti^III^ atoms form zigzag chains of edge-sharing [TiCl_4_O_2_] octa­hedra (Anokhina *et al.*, 2000 ▸). In other cases, the trivalent ions, such as Sc^III^ in ScNb_6_Cl_13_O_3_ (Cordier *et al.*, 1994 ▸) or Ti^III^ in Cs_2_Ti_3_Nb_12_Cl_27_O_8_ (Anokhina *et al.*, 2000 ▸), have a coordination number of five, defined by three O and two Cl ligands. In another case, the Gd^III^ atom in RbGdNb_6_Cl_18_ is octa­hedrally surrounded by six Cl ligands from six neighboring cluster units (Gulo *et al.*, 2023 ▸). In general, in the series of niobium oxychloride compounds containing octa­hedral Nb_6_ clusters, the crystallographic sites associated with trivalent cations are always fully occupied and are surrounded by Cl and O ligands (Gulo & Perrin, 2012 ▸). Only in Cs_2_GdNb_6_Cl_15_O_3_ and the isotypic uranium analogue, the trivalent cation occupy the center of a triangle formed by three adjacent cluster units and are bonded to nine ligands.

**Figure 4:**
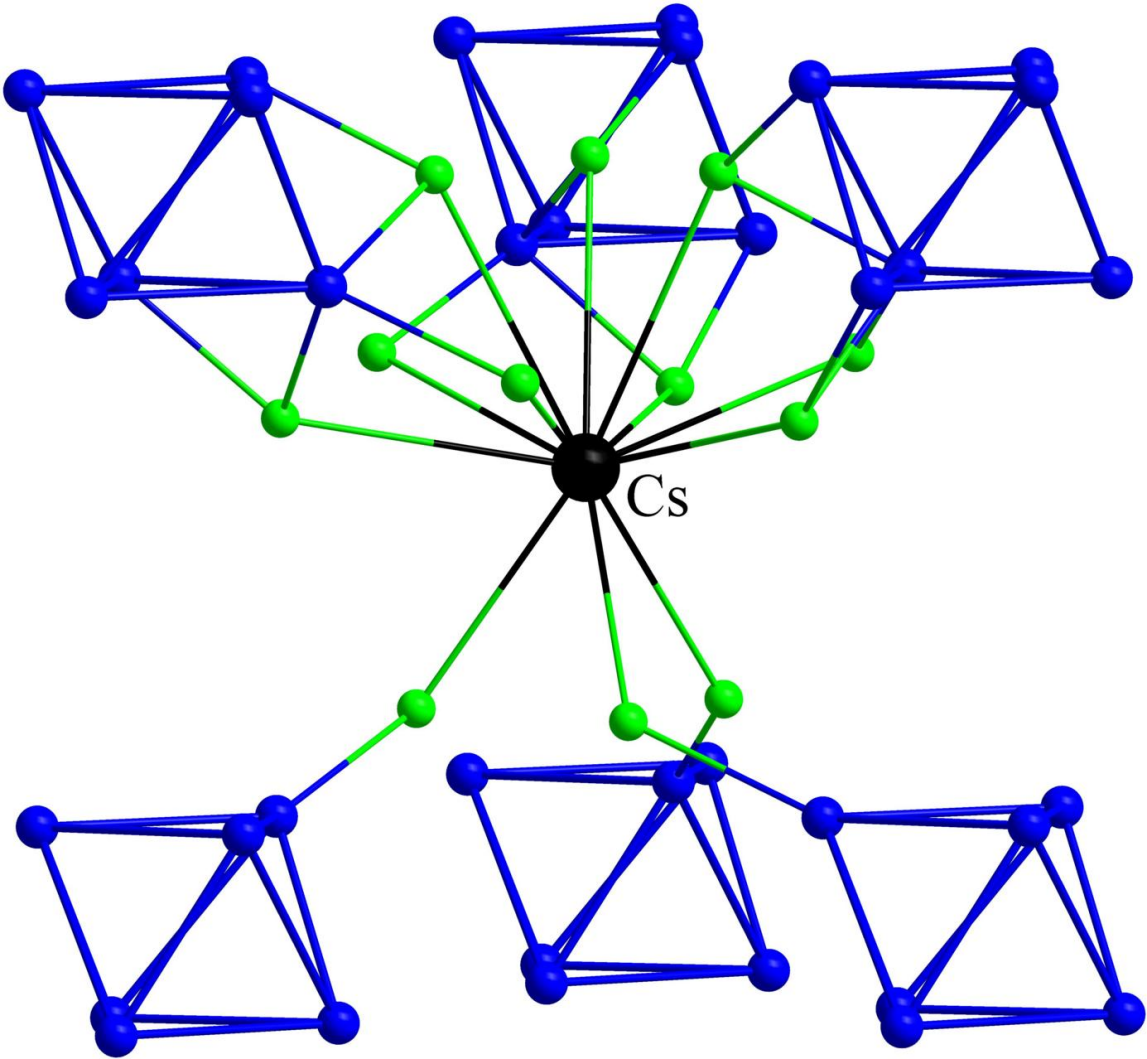
The environment of the Cs^I^ atom in Cs_2_GdNb_6_Cl_15_O_3_.

In Cs_2_GdNb_6_Cl_15_O_3_, the monovalent Cs^I^ atom is surrounded by six Nb_6_ clusters and coordinated by twelve Cl ligands (Fig. 4 ▸). The lengths of Cs—Cl bonds range from 3.5074 (8) to 3.9770 (6) Å. A similar environment around Cs is found in CsNb_6_Cl_12_O_2_ with Cs—Cl distances between 3.330 (5) and 3.862 (4) Å (Gulo & Perrin, 2000 ▸). In contrast, the Cs^I^ atom in Cs_2_LuNb_6_Cl_17_O is surrounded by four Nb_6_-clusters and is bonded to twelve chlorine ligands with Cs—Cl distances in the range 3.567 (1) to 3.619 (1) Å (Cordier *et al.*, 1996 ▸). In RbGdNb_6_Cl_18_ with its smaller monovalent cation Rb^I^, the coordination number is likewise 12. Here, the cation is surrounded also by four Nb_6_ clusters, and the Rb—Cl bond lengths range from 3.471 (1) Å to 3.557 (2) Å with an average of 3.512 Å (Gulo *et al.*, 2023 ▸). The sites of monovalent cations encountered in the crystal structures of oxychlorides with Nb_6_ clusters are always surrounded by Cl ligands with the exception of Cs_2_LuNb_6_Cl_17_O where an O atom statistically occupies a site among the twelve inner ligands defining the coordination environment of Cs. On the other hand, the sites associated with the (large) monovalent cation often show partial occupancy. For example, in Na_0.21_Nb_6_Cl_10.5_O_3_, the corresponding Na site has an occupancy of only 42.6% (Gulo & Perrin, 2002 ▸) and the three Cs sites in Cs_2_Ti_4_Nb_6_Cl_18_O_6_ have occupancies of 38.1%, 57.0% and 6.9% (Anokhina *et al.*, 2001 ▸).

The VEC in Cs_2_GdNb_6_Cl_15_O_3_ is 14 per cluster unit, as observed in most oxide (Köhler *et al.*, 1991 ▸) and oxychloride compounds (Gulo & Perrin, 2012 ▸). The number of O^*i*^ ligands per cluster can affect the VEC value. Compounds containing one O^*i*^ ligand (Cordier *et al.*, 1996 ▸) or two O^*i*^ ligands (Gulo & Perrin, 2000 ▸) exhibit VEC values of 16 and 15. However, niobium oxychloride compounds containing three or more O^*i*^ ligands per cluster unit have always a VEC of 14 per cluster unit.

## 3. Synthesis and crystallization

Cs_2_GdNb_6_Cl_15_O_3_ was prepared by solid-state reactions, starting from a stoichiometric mixture of CsCl (Prolabo, purity 99.5%), Gd_2_O_3_ (Rhône Poulenc), Nb_2_O_5_ (Merck, Optipur), NbCl_5_ (Ventron, purity 99.998%) and niobium powder (Ventron, purity 99.8%). A total of 300 mg of the mixture was mashed and then loaded in a silica tube under argon atmos­phere in a glove box. The silica tube sample was then sealed under vacuum condition. The sample was heated in a vertical heating furnace at 973 K for two days, followed by slow cooling to room temperature. Brown single crystals with a block-like form suitable for structural determination were obtained this way.

## 4. Refinement

Crystal data, data collection and structure refinement details are summarized in Table 1 ▸. The remaining maximum and minimum electron density peaks are located 0.19 Å from Nb and 0.54 Å from Cs, respectively.

## Supplementary Material

Crystal structure: contains datablock(s) I. DOI: 10.1107/S205698902300871X/wm5695sup1.cif

Structure factors: contains datablock(s) I. DOI: 10.1107/S205698902300871X/wm5695Isup2.hkl

CCDC reference: 2299097

Additional supporting information:  crystallographic information; 3D view; checkCIF report

## Figures and Tables

**Table 1:** Experimental details

Crystal data	
Chemical formula	Cs_2_GdNb_6_Cl_15_O_3_
*M*_r_	1560.28
Crystal system, space group	Trigonal, *P* 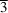 1*c*
Temperature (K)	293
*a*, *c* (Å)	9.1318 (1), 17.1558 (2)
*V* (Å^3^)	1238.95 (3)
*Z*	2
Radiation type	Mo *K*α
μ (mm^−1^)	9.83
Crystal size (mm)	0.08 × 0.07 × 0.05

Data collection	
Diffractometer	Nonius KappaCCD
Absorption correction	Multi-scan (*DENZO* and *SCALEPACK*; Otwinowski & Minor, 1997 ▸)
*T*_min_, *T*_max_	0.004, 0.017
No. of measured, independent and observed [*I* > 2σ(*I*)] reflections	7607, 2020, 1790
*R*_int_	0.026
(sin θ/λ)_max_ (Å^−1^)	0.833

Refinement	
*R*[*F* ^2^ > 2σ(*F* ^2^)], *wR*(*F* ^2^), *S*	0.029, 0.068, 1.15
No. of reflections	2020
No. of parameters	44
Δρ_max_, Δρ_min_ (e Å^−3^)	1.38, −1.35

Computer programs: *COLLECT* (Nonius, 1999 ▸), *SHELXT* (Sheldrick, 2015*a* ▸), *SHELXL* (Sheldrick, 2015*b* ▸), *DIAMOND* (Brandenburg, 2014 ▸), and *publCIF* (Westrip, 2010 ▸).

## supplementary crystallographic information

### Crystal data

**Table:**

Cs_2_GdNb_6_Cl_15_O_3_	*D*_x_ = 4.182 Mg m^−^^3^
*M**_r_* = 1560.28	Mo *K*α radiation, λ = 0.71073 Å
Trigonal, *P*31*c*	Cell parameters from 25 reflections
*a* = 9.1318 (1) Å	θ = 12–18°
*c* = 17.1558 (2) Å	µ = 9.83 mm^−^^1^
*V* = 1238.95 (3) Å^3^	*T* = 293 K
*Z* = 2	Block, brown
*F*(000) = 1398	0.08 × 0.07 × 0.05 mm

### Data collection

**Table:**

Nonius KappaCCD diffractometer	1790 reflections with *I* > 2σ(*I*)
Radiation source: fine-focus sealed tube	*R*_int_ = 0.026
ω scans	θ_max_ = 36.3°, θ_min_ = 2.4°
Absorption correction: multi-scan (DENZO and SCALEPACK; Otwinowski & Minor, 1997)	*h* = −15→15
*T*_min_ = 0.004, *T*_max_ = 0.017	*k* = −12→12
7607 measured reflections	*l* = −27→28
2020 independent reflections	

### Refinement

**Table:**

Refinement on *F*^2^	Primary atom site location: structure-invariant direct methods
Least-squares matrix: full	Secondary atom site location: difference Fourier map
*R*[*F*^2^ > 2σ(*F*^2^)] = 0.029	*w* = 1/[σ^2^(*F*_o_^2^) + (0.0258*P*)^2^ + 3.5848*P*] where *P* = (*F*_o_^2^ + 2*F*_c_^2^)/3
*wR*(*F*^2^) = 0.068	(Δ/σ)_max_ = 0.001
*S* = 1.15	Δρ_max_ = 1.38 e Å^−^^3^
2020 reflections	Δρ_min_ = −1.35 e Å^−^^3^
44 parameters	Extinction correction: SHELXL2018/3 (Sheldrick, 2015b), Fc^*^=kFc[1+0.001xFc^2^λ^3^/sin(2θ)]^-1/4^
0 restraints	Extinction coefficient: 0.00259 (19)

### Special details

**Table:**

Geometry. All esds (except the esd in the dihedral angle between two l.s. planes) are estimated using the full covariance matrix. The cell esds are taken into account individually in the estimation of esds in distances, angles and torsion angles; correlations between esds in cell parameters are only used when they are defined by crystal symmetry. An approximate (isotropic) treatment of cell esds is used for estimating esds involving l.s. planes.

### Fractional atomic coordinates and isotropic or equivalent isotropic displacement parameters (Å^2^)

**Table:**

	*x*	*y*	*z*	*U*_iso_*/*U*_eq_	
Cs	0.333333	0.666667	0.54654 (2)	0.02645 (9)	
Gd	0.666667	0.333333	0.750000	0.01649 (8)	
Nb	0.19768 (3)	0.01608 (3)	0.68239 (2)	0.01105 (7)	
Cl1	0.21283 (6)	−0.21283 (6)	0.750000	0.01643 (17)	
Cl2	0.01675 (8)	−0.20367 (8)	0.58643 (4)	0.01569 (12)	
Cl3	0.47649 (9)	0.07637 (10)	0.61760 (5)	0.02252 (15)	
O	0.3730 (3)	0.18650 (17)	0.750000	0.0142 (5)	

### Atomic displacement parameters (Å^2^)

**Table:**

	*U*^11^	*U*^22^	*U*^33^	*U*^12^	*U*^13^	*U*^23^
Cs	0.02466 (12)	0.02466 (12)	0.03002 (19)	0.01233 (6)	0.000	0.000
Gd	0.00919 (9)	0.00919 (9)	0.03108 (19)	0.00460 (5)	0.000	0.000
Nb	0.00836 (10)	0.00944 (10)	0.01540 (12)	0.00448 (7)	0.00090 (7)	0.00034 (7)
Cl1	0.0149 (3)	0.0149 (3)	0.0233 (4)	0.0103 (3)	0.0031 (3)	0.0031 (3)
Cl2	0.0144 (3)	0.0143 (3)	0.0180 (3)	0.0068 (2)	0.0007 (2)	−0.0028 (2)
Cl3	0.0177 (3)	0.0231 (3)	0.0298 (4)	0.0125 (3)	0.0090 (3)	0.0033 (3)
O	0.0092 (10)	0.0139 (9)	0.0179 (13)	0.0046 (5)	0.000	−0.0004 (8)

### Geometric parameters (Å, º)

**Table:**

Cs—Cl3^i^	3.5074 (8)	Gd—Cl3	3.0994 (8)
Cs—Cl3^ii^	3.5074 (8)	Gd—Cl3^viii^	3.0994 (8)
Cs—Cl3^iii^	3.5074 (8)	Gd—Cl3^vii^	3.0994 (8)
Cs—Cl3^iv^	3.5180 (8)	Gd—Cl3^ix^	3.0994 (8)
Cs—Cl3^v^	3.5180 (8)	Gd—Cl3^x^	3.0994 (8)
Cs—Cl3^vi^	3.5180 (8)	Gd—Cl3^iii^	3.0994 (8)
Cs—Cl2^i^	3.6948 (7)	Nb—O	1.9593 (19)
Cs—Cl2^iii^	3.6948 (7)	Nb—Cl1	2.4543 (7)
Cs—Cl2^ii^	3.6948 (7)	Nb—Cl2^ii^	2.4684 (7)
Cs—Cl1^iii^	3.9770 (6)	Nb—Cl2	2.4802 (7)
Cs—Cl1^ii^	3.9770 (6)	Nb—Cl3	2.5728 (7)
Cs—Cl1^i^	3.9770 (5)	Nb—Nb^x^	2.7686 (5)
Gd—O	2.322 (3)	Nb—Nb^xi^	3.0075 (4)
Gd—O^vii^	2.322 (3)	Nb—Nb^ii^	3.0075 (4)
Gd—O^iii^	2.322 (3)	Nb—Nb^xii^	3.0317 (5)
			
Cl3^i^—Cs—Cl3^ii^	108.589 (15)	O^iii^—Gd—Cl3^vii^	80.189 (14)
Cl3^i^—Cs—Cl3^iii^	108.589 (15)	Cl3—Gd—Cl3^vii^	72.21 (2)
Cl3^ii^—Cs—Cl3^iii^	108.589 (15)	Cl3^viii^—Gd—Cl3^vii^	98.06 (3)
Cl3^i^—Cs—Cl3^iv^	108.89 (2)	O—Gd—Cl3^ix^	80.189 (13)
Cl3^ii^—Cs—Cl3^iv^	76.72 (2)	O^vii^—Gd—Cl3^ix^	60.971 (13)
Cl3^iii^—Cs—Cl3^iv^	137.786 (19)	O^iii^—Gd—Cl3^ix^	130.969 (15)
Cl3^i^—Cs—Cl3^v^	76.72 (2)	Cl3—Gd—Cl3^ix^	98.06 (3)
Cl3^ii^—Cs—Cl3^v^	137.786 (19)	Cl3^viii^—Gd—Cl3^ix^	72.21 (2)
Cl3^iii^—Cs—Cl3^v^	108.89 (2)	Cl3^vii^—Gd—Cl3^ix^	121.94 (2)
Cl3^iv^—Cs—Cl3^v^	62.55 (2)	O—Gd—Cl3^x^	60.971 (13)
Cl3^i^—Cs—Cl3^vi^	137.786 (19)	O^vii^—Gd—Cl3^x^	130.969 (14)
Cl3^ii^—Cs—Cl3^vi^	108.89 (2)	O^iii^—Gd—Cl3^x^	80.189 (14)
Cl3^iii^—Cs—Cl3^vi^	76.72 (2)	Cl3—Gd—Cl3^x^	121.94 (3)
Cl3^iv^—Cs—Cl3^vi^	62.55 (2)	Cl3^viii^—Gd—Cl3^x^	72.21 (2)
Cl3^v^—Cs—Cl3^vi^	62.55 (2)	Cl3^vii^—Gd—Cl3^x^	160.38 (3)
Cl3^i^—Cs—Cl2^i^	61.822 (15)	Cl3^ix^—Gd—Cl3^x^	72.21 (2)
Cl3^ii^—Cs—Cl2^i^	54.828 (15)	O—Gd—Cl3^iii^	80.189 (14)
Cl3^iii^—Cs—Cl2^i^	148.77 (2)	O^vii^—Gd—Cl3^iii^	130.969 (14)
Cl3^iv^—Cs—Cl2^i^	69.076 (17)	O^iii^—Gd—Cl3^iii^	60.971 (13)
Cl3^v^—Cs—Cl2^i^	97.986 (17)	Cl3—Gd—Cl3^iii^	72.20 (2)
Cl3^vi^—Cs—Cl2^i^	131.531 (19)	Cl3^viii^—Gd—Cl3^iii^	121.94 (3)
Cl3^i^—Cs—Cl2^iii^	54.828 (15)	Cl3^vii^—Gd—Cl3^iii^	72.21 (2)
Cl3^ii^—Cs—Cl2^iii^	148.77 (2)	Cl3^ix^—Gd—Cl3^iii^	160.38 (3)
Cl3^iii^—Cs—Cl2^iii^	61.822 (15)	Cl3^x^—Gd—Cl3^iii^	98.06 (3)
Cl3^iv^—Cs—Cl2^iii^	131.531 (19)	O—Nb—Cl1	91.438 (10)
Cl3^v^—Cs—Cl2^iii^	69.076 (18)	O—Nb—Cl2^ii^	95.39 (2)
Cl3^vi^—Cs—Cl2^iii^	97.986 (17)	Cl1—Nb—Cl2^ii^	165.85 (2)
Cl2^i^—Cs—Cl2^iii^	116.649 (8)	O—Nb—Cl2	169.99 (6)
Cl3^i^—Cs—Cl2^ii^	148.77 (2)	Cl1—Nb—Cl2	85.583 (18)
Cl3^ii^—Cs—Cl2^ii^	61.822 (15)	Cl2^ii^—Nb—Cl2	85.58 (3)
Cl3^iii^—Cs—Cl2^ii^	54.828 (15)	O—Nb—Cl3	75.97 (6)
Cl3^iv^—Cs—Cl2^ii^	97.986 (17)	Cl1—Nb—Cl3	85.14 (2)
Cl3^v^—Cs—Cl2^ii^	131.531 (19)	Cl2^ii^—Nb—Cl3	84.52 (2)
Cl3^vi^—Cs—Cl2^ii^	69.076 (17)	Cl2—Nb—Cl3	94.24 (3)
Cl2^i^—Cs—Cl2^ii^	116.649 (8)	O—Nb—Nb^x^	45.05 (6)
Cl2^iii^—Cs—Cl2^ii^	116.649 (8)	Cl1—Nb—Nb^x^	94.843 (15)
Cl3^i^—Cs—Cl1^iii^	62.628 (14)	Cl2^ii^—Nb—Nb^x^	98.75 (2)
Cl3^ii^—Cs—Cl1^iii^	97.912 (19)	Cl2—Nb—Nb^x^	144.687 (16)
Cl3^iii^—Cs—Cl1^iii^	53.624 (14)	Cl3—Nb—Nb^x^	121.017 (19)
Cl3^iv^—Cs—Cl1^iii^	168.335 (15)	O—Nb—Nb^xi^	137.22 (6)
Cl3^v^—Cs—Cl1^iii^	119.844 (17)	Cl1—Nb—Nb^xi^	89.097 (14)
Cl3^vi^—Cs—Cl1^iii^	129.093 (15)	Cl2^ii^—Nb—Nb^xi^	94.227 (16)
Cl2^i^—Cs—Cl1^iii^	99.332 (16)	Cl2—Nb—Nb^xi^	52.396 (17)
Cl2^iii^—Cs—Cl1^iii^	51.659 (16)	Cl3—Nb—Nb^xi^	146.53 (2)
Cl2^ii^—Cs—Cl1^iii^	88.306 (13)	Nb^x^—Nb—Nb^xi^	92.291 (4)
Cl3^i^—Cs—Cl1^ii^	97.912 (19)	O—Nb—Nb^ii^	94.52 (4)
Cl3^ii^—Cs—Cl1^ii^	53.623 (14)	Cl1—Nb—Nb^ii^	139.044 (15)
Cl3^iii^—Cs—Cl1^ii^	62.629 (14)	Cl2^ii^—Nb—Nb^ii^	52.752 (17)
Cl3^iv^—Cs—Cl1^ii^	129.092 (15)	Cl2—Nb—Nb^ii^	93.982 (16)
Cl3^v^—Cs—Cl1^ii^	168.336 (16)	Cl3—Nb—Nb^ii^	135.56 (2)
Cl3^vi^—Cs—Cl1^ii^	119.844 (17)	Nb^x^—Nb—Nb^ii^	63.159 (10)
Cl2^i^—Cs—Cl1^ii^	88.305 (13)	Nb^xi^—Nb—Nb^ii^	60.0
Cl2^iii^—Cs—Cl1^ii^	99.332 (16)	O—Nb—Nb^xii^	93.78 (4)
Cl2^ii^—Cs—Cl1^ii^	51.660 (16)	Cl1—Nb—Nb^xii^	51.856 (14)
Cl1^iii^—Cs—Cl1^ii^	49.043 (19)	Cl2^ii^—Nb—Nb^xii^	139.534 (16)
Cl3^i^—Cs—Cl1^i^	53.623 (14)	Cl2—Nb—Nb^xii^	91.888 (19)
Cl3^ii^—Cs—Cl1^i^	62.629 (15)	Cl3—Nb—Nb^xii^	135.895 (19)
Cl3^iii^—Cs—Cl1^i^	97.912 (19)	Nb^x^—Nb—Nb^xii^	62.269 (11)
Cl3^iv^—Cs—Cl1^i^	119.843 (17)	Nb^xi^—Nb—Nb^xii^	54.570 (9)
Cl3^v^—Cs—Cl1^i^	129.092 (15)	Nb^ii^—Nb—Nb^xii^	87.296 (5)
Cl3^vi^—Cs—Cl1^i^	168.336 (16)	Nb^xii^—Cl1—Nb	76.29 (3)
Cl2^i^—Cs—Cl1^i^	51.659 (16)	Nb^xii^—Cl1—Cs^xiii^	142.343 (15)
Cl2^iii^—Cs—Cl1^i^	88.305 (13)	Nb—Cl1—Cs^xiii^	87.830 (6)
Cl2^ii^—Cs—Cl1^i^	99.333 (16)	Nb^xii^—Cl1—Cs^xiv^	87.831 (6)
Cl1^iii^—Cs—Cl1^i^	49.043 (19)	Nb—Cl1—Cs^xiv^	142.343 (15)
Cl1^ii^—Cs—Cl1^i^	49.043 (19)	Cs^xiii^—Cl1—Cs^xiv^	122.73 (2)
O—Gd—O^vii^	120.0	Nb^xi^—Cl2—Nb	74.85 (2)
O—Gd—O^iii^	120.0	Nb^xi^—Cl2—Cs^xiii^	146.20 (3)
O^vii^—Gd—O^iii^	120.000 (1)	Nb—Cl2—Cs^xiii^	94.06 (2)
O—Gd—Cl3	60.971 (13)	Nb—Cl3—Gd	88.01 (2)
O^vii^—Gd—Cl3	80.190 (14)	Nb—Cl3—Cs^xiii^	96.94 (2)
O^iii^—Gd—Cl3	130.969 (14)	Gd—Cl3—Cs^xiii^	146.22 (3)
O—Gd—Cl3^viii^	130.969 (14)	Nb—Cl3—Cs^v^	126.46 (3)
O^vii^—Gd—Cl3^viii^	80.189 (14)	Gd—Cl3—Cs^v^	100.30 (2)
O^iii^—Gd—Cl3^viii^	60.971 (13)	Cs^xiii^—Cl3—Cs^v^	103.28 (2)
Cl3—Gd—Cl3^viii^	160.38 (3)	Nb^x^—O—Nb	89.91 (11)
O—Gd—Cl3^vii^	130.969 (15)	Nb^x^—O—Gd	135.05 (6)
O^vii^—Gd—Cl3^vii^	60.971 (13)	Nb—O—Gd	135.05 (6)

Symmetry codes: (i) *x*, *y*+1, *z*; (ii) −*y*, *x*−*y*, *z*; (iii) −*x*+*y*+1, −*x*+1, *z*; (iv) *y*, −*x*+*y*+1, −*z*+1; (v) −*x*+1, −*y*+1, −*z*+1; (vi) *x*−*y*, *x*, −*z*+1; (vii) −*y*+1, *x*−*y*, *z*; (viii) −*y*+1, −*x*+1, −*z*+3/2; (ix) −*x*+*y*+1, *y*, −*z*+3/2; (x) *x*, *x*−*y*, −*z*+3/2; (xi) −*x*+*y*, −*x*, *z*; (xii) −*y*, −*x*, −*z*+3/2; (xiii) *x*, *y*−1, *z*; (xiv) −*y*+1, −*x*, −*z*+3/2.
